# Pauci-Immune Crescentic Glomerulonephritis: An ANCA-Associated Vasculitis

**DOI:** 10.1155/2015/402826

**Published:** 2015-11-25

**Authors:** Rafeel Syed, Amina Rehman, Gautam Valecha, Suzanne El-Sayegh

**Affiliations:** Division of Nephrology, Staten Island University Hospital, 475 Seaview Avenue, Staten Island, NY 10305, USA

## Abstract

*Rapidly progressive glomerulonephritis* (RPGN) is a syndrome signified by a precipitous loss of renal function, with features of glomerulonephritis including dysmorphic erythrocyturia and glomerular proteinuria. RPGN is associated with extensive crescent formation, and, thus, the clinical term RPGN is often used interchangeably with the pathologic term* crescentic glomerulonephritis* (CGN). From an immunopathologic standpoint, primary RPGN is divided into* pauci-immune GN* (PICG), anti-GBM GN, and immune complex GN. PICG, the most common etiology of primary RPGN, refers to a necrotizing glomerulonephritis with few or no immune deposits by immunofluorescence (IF) or electron microscopy (EM). In most patients, pauci-immune CGN is a component of a systemic small vessel vasculitis such as granulomatosis with polyangiitis (GPA). Approximately 90% of patients with PICG have circulating ANCA antibodies, leading to the nomenclature* ANCA-associated vasculitis* (AAV). Recent research has identified several other antibodies associated with PICG, which is now understood to be a complex spectrum of disease with considerable overlap in terms of clinical phenotype and outcomes. In addition, several genetic and environmental factors have recently been implicated in the pathogenesis of this disorder. With new prognostic classifications, enhanced understanding of immunopathologic mechanisms, and novel treatment paradigms, clinical and experimental interest in PICG remains high.

## 1. Introduction: Epidemiology and Clinical Outcomes

At a population level, little is known about the epidemiology and outcome of pauci-immune GN. PICG represents up to 80% of cases of RPGN, the incidence of which is estimated to be 7–10 cases per million people per year in the United States [1]. Pauci-immune GN (PICG) has a predilection for whites compared to blacks, with roughly equal representation in men and women [1]. Interestingly, GPA is more common in cooler climates, whereas MPA is more frequent in warmer climates. AAV in Asia is more often associated with MPO-ANCA than with PR3-ANCA [2]. Without treatment, PICG has a 1-year mortality of 80%. With aggressive immunosuppression, however, the 5-year survival is up to 75% [3]. Older age, dialysis dependency, and pulmonary hemorrhage all worsen the chances of survival. For instance, irreversible, dialysis-dependent renal failure lowers the 5-year survival rate to 35%. From a renal outcome standpoint, about 25% of patients progress to ESRD [4]. The best predictor of renal outcomes is the initial serum creatinine, as well as the extent of renal injury and fibrosis on biopsy. Although remission can be induced in most patients, about 40% of patients relapse, indicating the need for close monitoring [1]. Here, we present a case of renal-limited PICG presenting with dialysis-dependent renal failure. The ensuing discussion aims to detail the pathophysiology of PICG, while highlighting possible avenues for future scientific inquiry.

## 2. Nomenclature and Classification 

Though descriptive and current, the classification “pauci-immune” glomerulonephritis can be somewhat incomplete and misleading. Historically, the term was coined to characterize the lack of linear immunoglobulin (type I) or immune complex (type II) deposition on immunofluorescence [1]. This, however, does not imply that the immune system is not involved in the pathogenesis of the disease process. ON the contrary, pauci-immune GN is a classically autoimmune renal disease and is thus treated as such.

Despite efforts to simplify the classification system, the term “pauci-immune glomerulonephritis” represents intricate and overlapping “spectrum” of disease processes. We know that about 10% of the cases in the pathologic continuum of PICG are ANCA negative despite similar clinical features and renal biopsy findings as compared to ANCA-positive cases [1]. In addition, although pauci-immune necrotizing GN typically occurs in association with involvement of other organs in both GPA and MPA, some patients present with a* renal-limited*, ANCA-positive vasculitis. Up to 80% of these patients have MPO-ANCA positivity, while the remaining have anti-PR3-ANCA antibodies [5]. The patient presented in this review has anti-PR3-ANCA, which upon remission carries a higher relapse rate and an overall worse prognosis. Renal-limited vasculitis is considered to be part of the spectrum of GPA and MPA because the histopathologic findings of the two conditions are often indistinguishable [6]. Clinically, patients with renal-limited vasculitis are more likely to have glomerulosclerosis on renal biopsy than those with GPA or MPA, since patients without extrarenal manifestations probably present later in the course of their disease [6]. Adding to the complexity of the clinical phenotype, some patients have small, dispersed immune deposits in the mesangium and glomerular capillary wall when assessed by electron microscopy. This, in the proper clinical setting and positive ANCA antibodies, is still classified as “pauci-immune” glomerulonephritis [7]. Lastly, the entity of “double-antibody” positive disease must also be mentioned, as it includes patients with features of both ANCA-positive RPGN and anti-GBM disease [8].

## 3. Case

**Figure 1:**
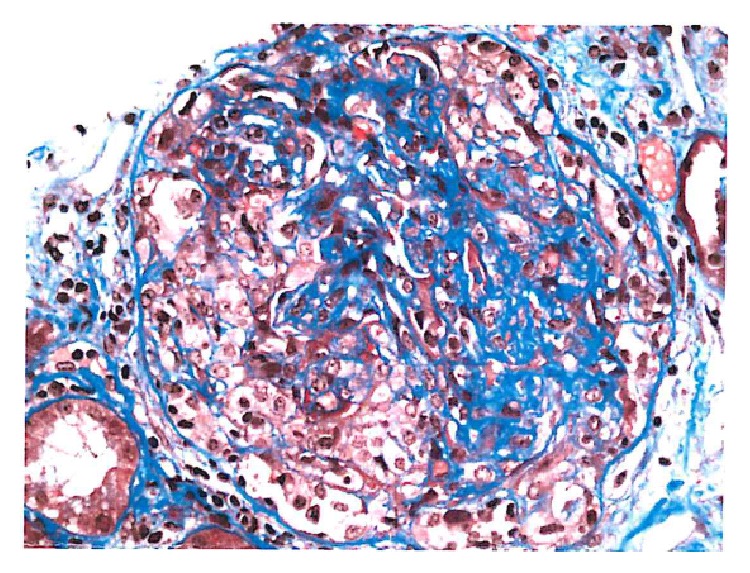
Cellular crescent.

**Figure 2:**
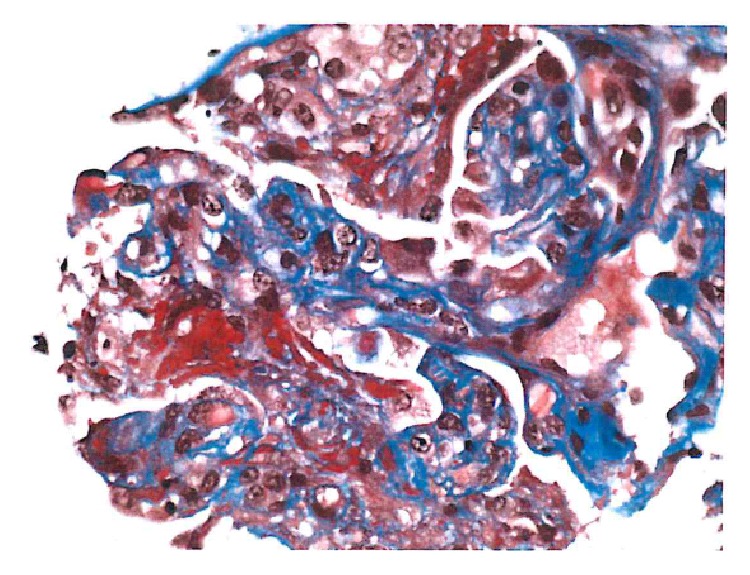
Fibrinoid necrosis.

**Figure 3:**
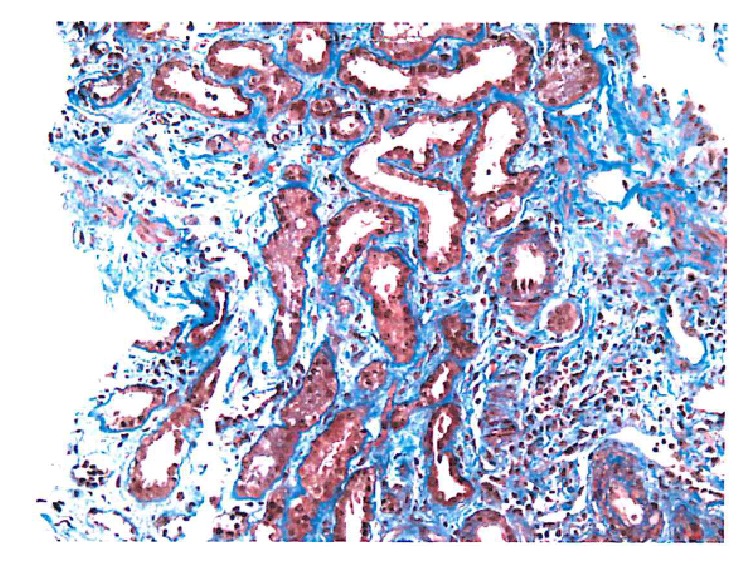
Mild tubular atrophy.

63-year-old Caucasian female with past medical history of diabetes mellitus type II for 7 years (without retinopathy or neuropathy), hypertension, and hypothyroidism presented with acute kidney injury in March of 2015. Patient was edematous on exam and had a peak serum creatinine of 7.2 mg/dL. Initial findings were also significant for hyperkalemia and hypertension. She was initiated on hemodialysis for hyperkalemia, volume overload, and uremia. Medications prior to admission included occasional NSAID use, metformin, and ACEI, all of which were initially held. Urinalysis showed 100 mg/dL of protein with >5 RBC/HPF, without casts on microscopy. 24-hour urine was quantified for protein and determined to be 3 grams. ANA, anti-DNA, complement levels, hepatitis and HIV serologies, ASLO, and anti-GBM were all negative. ANCA serology yielded anti-myeloperoxidase (MPO) antibody level of <1 and an elevated anti-proteinase 3 (PR3) antibody level at 54.3. SPEP/UPEP was noncontributory. On renal ultrasound, kidneys were of the size of approximately 11 cm and 14 cm, without evidence of chronic renal disease, atrophy, or hydronephrosis. Patient underwent a kidney biopsy, which showed mild chronicity secondary to hypertension-related nephrosclerosis, along with mild tubular atrophy and interstitial fibrosis (Figure 3). Acute histological findings on biopsy were significant for focal segmental necrotizing (Figure 2) and diffuse crescentic glomerulonephritis (Figure 1), pauci-immune type, with moderate-to-severe activity and mild chronicity. In addition, diffuse acute tubular injury was seen with focal red blood cell casts. No changes secondary to diabetes were seen on the biopsy. Patient received induction therapy with cyclophosphamide and intravenous corticosteroids and maintenance immunosuppression with azathioprine and oral prednisone. She remained dialysis-dependent and developed untoward side effects to azathioprine therapy, necessitating its discontinuation. She is currently considered to have ESRD.

## 4. Histopathological Classification

Renal biopsy is of critical importance in the diagnosis of PICG and RPGN in general. Even in patients with predominant lung involvement, kidney biopsy may be preferred as it is associated with a higher diagnostic yield and lower procedural risk [9]. The distinctive pathological features associated with PICG are necrosis of the capillary loops, extracapillary proliferation with crescent formation, periglomerular and interstitial infiltrates, necrotizing arteritis, and absence or paucity of immune deposits [10].

A crescent is characterized by extracapillary proliferation within a glomerulus, either partially or completely filling up Bowman's space. It is composed of proliferating parietal epithelial cells, podocytes, macrophages, and fibroblasts. Crescent formation is stimulated by the entry of fibrin and other plasma proteins from the capillary lumens following the rupture of the glomerular basement membrane (GBM). This is followed by the migration of T cells, macrophages, and other inflammatory cells to the site of injury, accelerating cytokine release and tissue injury. These “cellular” crescents may finally evolve into fibrocellular or fibrous crescents with increased extracellular matrix and collagen deposition. Thus, the presence of GBM rupture in a sclerosed glomerulus should raise suspicion for previous crescentic glomerular injury.

The arrangement and the degree of crescent formation as well as the presence of sclerosis play a significant role in determining the clinical course of PICG [11, 12]. Berden et al. recently presented a histopathologic classification system developed by the international working group of renal pathologists. They performed a validation study on 100 renal biopsies from patients with clinically and histopathologically confirmed diagnosis of ANCA-associated glomerulonephritis. The authors divided renal biopsies into four histological categories as seen under light microscopy: focal, crescentic, mixed, and sclerotic based on the proportion of normal, cellular, crescentic, and sclerotic glomeruli present. Their analysis showed that this classification system has good prognostic value in terms of renal outcome at 1 and 5 years. Renal survival at 1 year was 93% for patients whose renal biopsies were classified as focal at the time of diagnosis, 84% for patients whose biopsies were classified as crescentic, 69% for patients whose biopsies were classified as mixed, and 50% for patients whose biopsies were classified as sclerotic. Renal survival percentages at 5 years were 93% (focal), 76% (crescentic), 61% (mixed), and 50% (sclerotic). In the sclerotic category, renal survival at 7 years was only 25%. Several validation studies out of Japan, China, Australia, United States, Netherlands, and Turkey have further confirmed that the outlined histopathological classification of ANCA-associated glomerulonephritis is of predictive value in terms of renal outcome [13–15]. This was especially true for the outlier categories of focal and sclerotic glomerular injuries.

Although in PICG, by definition, there is a paucity of immune deposits on immunofluorescence microscopy and electron microscopy, a significant proportion of cases may indeed show Ig deposits on histopathological examination [7, 16, 17]. Neumann et al. [17] showed that the presence of Ig deposits resulted in significantly more proteinuria and may be associated with worse prognosis. This can be explained by the hypothesis that ANCA and immune complexes may act synergistically to produce a more severe injury at the glomerulus. It is also important to note that experimental animal models have not convincingly ruled out the presence of immune deposits in ANCA-associated glomerulonephritis [18, 19]. Rats preimmunized with human MPO-ANCA that were subsequently perfused with lysosomal extracts and H2O2 developed CGN and also showed IgG and C3 deposition early in the course of the disease process. Moreover, the degree of histologic injury was proportional to the amount of IgG immune deposits. Interestingly, the Ig deposits could not be detected later in the disease course, suggesting that the presence of immune complexes may be time dependent and not seen by the time most patients are biopsied. Finally, there are also reports of ANCA-associated glomerulonephritis complicating immune complex related diseases such as IgA nephropathy, membranous glomerulonephritis, and postinfectious glomerulonephritis [7, 20–22]. Thus, it is important to test for ANCA even in cases with immune deposits on renal histology (type II lesions).

## 5. Pathophysiology

Despite the paucity of immune deposits, PICG is a classically immune mediated disease process. Most of the* in vitro, in vivo,* and clinical studies point to the involvement of ANCA in the pathogenesis of this disease [23–26]. One example of direct evidence implicating the role of ANCA comes from a case report of neonatal microscopic polyangiitis secondary to the transplacental transfer of MPO-ANCA [24]. This finding, though interesting, needs further substantiation in future studies. In addition, several animal models of ANCA-associated renal disease have also been described. Xiao et al. [25] injected splenocytes from MPO-immunized mice into both B and T cell depleted mice and wild type mice. The recipient mice developed pauci-immune necrotizing crescentic glomerulonephritis and hemorrhagic pulmonary capillaritis, almost identical to the histopathology and phenotype of human MPO-ANCA-associated vasculitis. Transfer of IgG alone from MPO-immunized mice resulted in pauci-immune focal necrotizing CGN in the recipient, clearly demonstrating the role of anti-MPO antibodies. Although* in vivo* evidence supporting the role of MPO-ANCA is well documented, the results have not yet been convincingly replicated for PR3-ANCA disease in animal models [27]. Some experts have thus questioned the role of PR3 antibodies in the induction of ANCA disease and have instead proposed other mechanisms such as T cell mediated inflammation.

Anti-neutrophil cytoplasmic autoantibodies (ANCA) are specific for proteins in the cytoplasm of neutrophils and monocytes. The major target antigens in patients with vasculitis and glomerulonephritis are myeloperoxidase (MPO) and proteinase 3 (PR3). After binding with the target antigens PR3 or MPO, ANCA results in the activation of neutrophils and monocytes once the cells are primed with low doses of cytokines such as tumor necrosis factor alpha (TNF-*α*), interleukin-1, and interleukin-18 [26, 28, 29]. There are priming results in the surface expression of PR3 and MPO, allowing interaction with ANCA. Neutrophil activation is mediated by the engagement of both Fc*γ* and Fab′2 receptors [30, 31]. In particular, the Fc*γ* IIa and IIIb receptors are involved. ANCA-mediated activation of neutrophils results in the production of reactive oxygen species and the release of lytic enzymes such as elastase that are injurious to the endothelial cells [28, 32, 33]. ANCA also stimulates the release of neutrophil extracellular traps (NETs) that are composed of chromatin fibers and autoantigens including MPO and PR3 [34, 35]. NETs can damage endothelial cells and entire glomeruli and may also be involved in propagating the ANCA autoimmune response [34]. In addition, it has become clear that the alternative pathway of complement activation plays a role in mediating tissue injury in PICG [36, 37]. Activated neutrophils release various factors such as properdin, which activate the alternate pathway with the generation of C3. Properdin, a molecule stored in neutrophilic granules, is released when C5a primes the activation of neutrophils. This optimizes the interaction with ANCA and helps activate the alternate complement pathway. All this sets off a cascade of events that augment neutrophil recruitment and activation, resulting in the widespread necrotizing lesions seen in AAV [37]. It is important to note that ANCA antigen specificity appears to be more closely associated with disease phenotype and prognosis than with the clinical diagnosis of GPA or MPA [38, 39]. PR3-ANCA is associated with granulomatous inflammation, more extensive extrarenal involvement, and a higher relapse rate. In contrast, MPO-ANCA is more frequent in renal-limited disease, displays more kidney scarring, and carries an overall worse renal prognosis.

Antibodies other than ANCA have also been studied in patients with PICG. Kain et al. demonstrated that a new subtype of ANCA, lysosomal membrane protein 2 (LAMP-2) antibodies, are more prevalent than MPO and PR3 antibodies in patients with biopsy proven pauci-immune focal necrotizing glomerulonephritis (FNGN) [40]. They also showed the development of characteristic lesions in the kidneys of rats after injecting them with LAMP-2 antibodies and apoptosis of human endothelium* in vitro* by monoclonal antibody to human LAMP-2 (H4B4). A subsequent study performed by the same group not only showed the high prevalence of LAMP-2 antibodies in patients with PICG but also demonstrated that they become undetectable with treatment and frequently detectable during a relapse [41], which has obvious clinical implications. However, another study could not establish a relationship between LAMP-2 antibodies and PICG [42]. Some investigators have suggested that this difference in the results can be attributed to the patient selection criteria and the assays used [43]. Regardless, these observations are intriguing and merit further study. Finally, anti-plasminogen antibodies found in some patients with AAV have been linked to increased susceptibility to thromboembolic events and greater severity of renal and systemic disease [44, 45].

Several environmental factors have now been associated with the pathogenesis of PICG. Microbial factors play a role in initiating the autoimmune response via their mimicry of host antigens [40]. Previous studies have demonstrated that patients with GPA are often chronic nasal carriers of* Staphylococcus aureus* and that antimicrobial therapy may decrease relapses [46, 47].* S. aureus* infection may also enhance the ability of the neutrophils to form NETs in small vessel vasculitis [34]. More recent evidence has identified LAMP-2 epitope with 100% homology to fimbrial adhesin (FimH) seen in Gram-negative bacteria [48]. It has been hypothesized that immune response to FimH as seen in urosepsis triggers anti-LAMP-2 antibodies due to molecular mimicry.

Another line of evidence implicating the role of microbial factors comes from* in vivo* animal studies evaluating the role of toll-like receptors (TLRs) in the pathogenesis of PICG [49, 50]. TLRs are a class of receptors present on leukocytes that are capable of recognizing infectious organisms and play an important role in generating innate immune response. TLR4 ligation has been found to play a role in AAV induction. It results in stimulation of T helper 17 (Th17) and Th1 responses via TLR2 and TLR9 activation, respectively [49].

Several nonmicrobial environmental factors have also been linked to PICG. Silica is a known risk factor for ANCA [51, 52]. Certain drugs such as propylthiouracil, hydralazine, D-penicillamine, cefotaxime, minocycline, anti-TNF agents, phenytoin, and certain psychoactive agents are also known to cause ANCA-associated disease [53–56].

The role of autoimmune mechanisms in pauci-immune CGN is also supported by another theory that states that the initial immune response is generated against the complementary PR3 (cPR3) peptide (generated by antisense DNA strand of PR3) or a mimic of cPR3 rather than against PR3 peptide [57–59]. Patients with PR3-ANCA disease harbor not only circulating antibodies against sense PR3 peptides but also a separate set of antibodies against antisense complementary PR3 peptides (anti-cPR3). The immune response against cPR3 results in the production of anti-cPR3 antibodies which in turn generate anti-idiotypic antibodies that recognize not only the idiotope on the anti-cPR3 antibodies but also the epitope on the sense PR3 peptide (original autoantigen). Interestingly, a number of pathogens such as* S. aureus*, Ross River virus, and* Entamoeba histolytica* have sequences similar to cPR3 peptide, thus making them capable of generating the autoimmune response [58]. This provides another link between infection and autoimmunity in PICG.

The role of genetic factors in the pathogenesis of PICG is evident from the fact that not every individual with environmental risk factors develops the disease. It is also observed that AAV is more common in Caucasian population. The understanding of genetics in AAV has greatly improved after the publication of two genome-wide association studies (GWASs) [60, 61]. The goal of GWASs is to interrogate a large number of single-nucleotide polymorphisms (SNPs) that cover a big portion of the human genome. Interestingly, the three main AAV subtypes are associated with distinct HLA variants, that is, granulomatosis with polyangiitis (Wegener's) GPA with* HLA-DP1*, microscopic polyangiitis (MPA) with* HLA-DQ* and eosinophilic GPA (Churg-Strauss) with* HLA-DRB4*. Patients with GPA are also known to have a higher proportion of neutrophils with increased surface expression of PR3 [62]. GWASs also revealed that polymorphic variants of genes encoding PR3 and its main inhibitor, alpha-1 antitrypsin, are highly associated with GPA especially with PR3-ANCA positivity regardless of the clinical diagnosis. Candidate gene approach studies have shown associations with other variants involved in AAV, such as those belonging to the* CTLA-4* and* PTPN22* genes. These results have not yet been replicated in larger studies [63–65].

Also, it is important to discuss the role of T cell mediated immune response in PICG. It is widely known that CD4+ helper T cells with interferon-gamma secretion are involved in granuloma formation. Granulomas are seen mainly in PR3 AAV and not in MPO AAV. TH17 cells play a major role in causing granulomatous inflammation and vascular injury in PR3 AAV [66]. Morgan et al. have also shown diminished number and functional impairment of regulatory T cells (Tregs) in GPA [67]. Finally, according to some studies, patients with ANCA-mediated vasculitis have impaired functioning of Tregs along with the presence of effector T cells that are resistant to suppression by Tregs [68].

In addition to autoantibody production, B cells also have the ability to present antigens to T cells and may modulate T cell responses in small vessel vasculitis. Regulatory B cells (Bregs) are a distinct B cell subset cells that regulate the action of the T cell population by secreting IL-10. Wilde et al. demonstrated that there is a reduction in the number of Bregs in both active and quiescent AAV [69]. The study also revealed a positive correlation between Breg and Treg in quiescent AAV and that Th1 cell suppression by Breg may be inadequate in active AAV.

Recently, the pathogenic mechanisms of tissue damage in GPA, resulting in saddle nose deformity and orbital destruction, have been further elucidated [38, 70]. Fibroblasts lining bone and cartilage have been acknowledged to play the role of activated effector cells of tissue destruction in GPA. In the first* in vivo* model of GPA, Kesel et al. subcutaneously coimplanted the biopsy specimens from patients with active GPA or sinusitis (controls) with healthy allogeneic human nasal cartilage into immunodeficient mice [70]. GPA implants showed massive destruction of the coimplanted human cartilage, whereas cartilage destruction was only marginal in control samples. Further, cartilage destruction was mediated by human fibroblasts and could be controlled, similar to GPA in humans, with corticosteroids. In addition, in fibrotic lung disease, NETs were shown to promote differentiation and function of fibroblasts, indicating that NETs are involved not only in inflammation but also in the resulting fibrosis [38, 71].

## 6. Clinical Manifestations and Natural History

Notwithstanding a slightly higher specificity, ANCA is to pauci-immune RPGN as anti-DNA is to systemic lupus erythematosus. Microscopic polyangiitis (MPA), granulomatosis with polyangiitis (GPA), eosinophilic granulomatosis with polyangiitis (EGPA or Churg-Strauss syndrome), and renal-limited pauci-immune crescentic GN (PICG) are all associated with ANCA. The term “pauci-immune” RPGN encapsulates all of these conditions. Generalized nonspecific manifestations of systemic inflammatory disease, such as fever, malaise, anorexia, weight loss, myalgia, and arthralgia, can be seen in any of these diseases. The clinical manifestations of each entity vary based on the chronicity and sites of involvement. Patients without systemic involvement may present later in the course of their disease. Granulomatous involvement, particularly of the upper respiratory tract, is a characteristic feature of GPA, but not of MPA; eosinophilia, asthma, and atopy typically occur in EGPA. In each of these conditions, patients can have pulmonary hemorrhage caused by hemorrhagic alveolar capillaritis, which has treatment and prognostic implications [2]. As mentioned previously, the renal failure seen in PICG usually presents clinically as RPGN. It should be noted, though, that patients with GPA or MPA could also present with subacute or chronic nephritis. In one study assessing the clinical characteristics of pauci-immune crescentic GN, a cohort of more than 300 patients evaluated at renal biopsy had a mean age of 56 ± 20 years (range 2 to 92 years), male-to-female ratio of 1.0 : 0.9, mean serum creatinine concentration of 6.5 ± 4.0 mg/dL (range 0.8 to 22.1 mg/dL), and proteinuria of 1.9 ± 3.0 g/day (range 0.1 to 18 g/day) [2]. Older age, higher serum creatinine concentration at presentation, pulmonary hemorrhage, and dialysis-dependent renal failure correlate with an overall poor prognosis. Respiratory tract disease and PR3-ANCA are predictors of higher relapse rates [2]. Regardless of the category of ANCA disease though, the best clinical predictor of renal outcome is the glomerular filtration rate (GFR) at the time of diagnosis [1]. Interestingly, in one of the recent studies, the presence of ear, nose, and throat (ENT) involvement in AAV patients was found to be associated with lesser degree of interstitial fibrosis and tubular atrophy on kidney biopsy and better renal function. These results indicate that there may be different phenotypes of AAV defined by ENT involvement [71].

## 7. Conclusion

Despite significant advances in our understanding of the immune system, issues of incomplete efficacy, treatment toxicity, and infectious complications persist. With targeted approaches to immunosuppression and newer biologic agents modulating B and T cell function, the future holds promise in terms of greater treatment efficacy with an improved side effect profile. We believe that future studies will highlight the intricacies of the clinical and pathological spectrum of PICG, while proposing novel mechanisms for inducing and maintaining remission. Based on current guidelines, for patients in complete remission and without extrarenal manifestations for at least 12 months, renal transplant should not be delayed. ANCA positivity is not considered a contraindication for transplantation. However, no prospective data are available to assess the likelihood of recurrent ANCA vasculitis after kidney transplantation. In addition, the impact of disease activity or ANCA positivity at the time of transplantation on patient outcome is unclear. Future studies must be designed to gather prospective data that helps answer these questions.
